# Impact of Home Environment Interventions on the Risk of Influenza-Associated ARI in Andean Children: Observations from a Prospective Household-Based Cohort Study

**DOI:** 10.1371/journal.pone.0091247

**Published:** 2014-03-12

**Authors:** Philip J. Budge, Marie R. Griffin, Kathryn M. Edwards, John V. Williams, Hector Verastegui, Stella M. Hartinger, Daniel Mäusezahl, Monika Johnson, Jennifer M. Klemenc, Yuwei Zhu, Ana I. Gil, Claudio F. Lanata, Carlos G. Grigalva, for the RESPIRA PERU Group

**Affiliations:** 1 Division of Infectious Diseases, Department of Internal Medicine, Vanderbilt University, Nashville, Tennessee, United States of America; 2 Department of Health Policy, Vanderbilt University, Nashville, Tennessee, United States of America; 3 Vanderbilt Vaccine Research Program, Division of Infectious Diseases, Department of Pediatrics, Vanderbilt University, Nashville, Tennessee, United States of America; 4 Department of Pediatrics, Vanderbilt University, Nashville, Tennessee, United States of America; 5 Department of Pathology, Microbiology, and Immunology, Vanderbilt University, Nashville, Tennessee, United States of America; 6 Instituto de Investigación Nutricional, Lima, Peru; 7 Swiss Tropical and Public Health Institute, and University of Basel, Basel, Switzerland; 8 Department of Biostatistics, Vanderbilt University, Nashville, Tennessee, United States of America; University Hospital San Giovanni Battista di Torino, Italy

## Abstract

**Background:**

The Respiratory Infections in Andean Peruvian Children (RESPIRA-PERU) study enrolled children who participated in a community-cluster randomized trial of improved stoves, solar water disinfection, and kitchen sinks (IHIP trial) and children from additional Andean households. We quantified the burden of influenza-associated acute respiratory illness (ARI) in this household-based cohort.

**Methods:**

From May 2009 to September 2011, we conducted active weekly ARI surveillance in 892 children age <3 years, of whom 272 (30.5%) had participated in the IHIP trial. We collected nasal swabs during ARI, tested for influenza and other respiratory viruses by RT-PCR, and determined influenza incidence and risk factors using mixed-effects regression models.

**Results:**

The overall incidence of influenza-associated ARI was 36.6/100 child-years; incidence of influenza A, B, and C was 20.5, 8.7, and 5.2/100 child-years, respectively. Influenza C was associated with fewer days of subjective fever (median 1 vs. 2) and malaise (median 0 vs. 2) compared to influenza A. Non-influenza ARI also resulted in fewer days of fever and malaise, and fewer healthcare visits than influenza A-associated ARI. Influenza incidence varied by calendar year (80% occurred in the 2010 season) and IHIP trial participation. Among households that participated in the IHIP trial, influenza-associated ARI incidence was significantly lower in intervention than in control households (RR 0.40, 95% CI: 0.20–0.82).

**Conclusions:**

Influenza burden is high among Andean children. ARI associated with influenza A and B had longer symptom duration and higher healthcare utilization than influenza C-associated ARI or non-influenza ARI. Environmental community interventions may reduce influenza morbidity.

## Introduction

Influenza virus is a major cause of childhood morbidity, generating three to five million severe illnesses and up to 500,000 deaths annually worldwide. [1] Seasonal epidemics are caused by both influenza A and B, which are the targets of annual influenza vaccination. Influenza C also causes acute respiratory illness (ARI), but does not contribute significantly to seasonal epidemics and is not included in influenza vaccines [2].

Over the past decade, the burden of influenza among children has been increasingly recognized in both developing [3]–[5] and developed nations [6]. The study of Respiratory Infections in Andean Peruvian Children (RESPIRA-PERU), conducted from May 2009 to September 2011, documented high rates of acute respiratory illness (ARI) in Andean Peruvian children [7], [8].

Influenza is unique among viral respiratory pathogens in that illness can be prevented or attenuated by vaccination, and understanding the burden and temporal circulation of influenza is important for the implementation of vaccination policy. Using data from RESPIRA-PERU, we sought to determine the incidence of laboratory-confirmed influenza in Andean children, describe the clinical characteristics of these infections, and identify risk factors for influenza acquisition.

## Methods

### Ethics statement

The study protocol was approved by the Vanderbilt Institutional Review Board and by the ethics committee of the Instituto de Investigación Nutricional. Written informed consent was obtained from a parent or guardian of all children involved in this study.

### RESPIRA-PERU study

RESPIRA-PERU, a prospective household-based study of ARI conducted in the high-altitude (1500–4000 meters) province of San Marcos, Cajamarca, Peru has been previously described [7], [8]. Families with children <3 years of age (including newborns) were enrolled between March, 2009 and August, 2011 and were under active ARI surveillance from May, 2009 through September, 2011. To capture as many ARI as possible, a highly sensitive ARI definition (cough or fever) was employed. After obtaining signed informed consent, field workers obtained baseline demographic and socioeconomic information and permission to access vaccination and healthcare records for enrolled children. Once enrolled, children were followed until their 3^rd^ birthday, withdrawal of consent, loss to follow-up, death, or study end, whichever came first. Field-workers visited the home of each enrolled child weekly, interviewed the parent or guardian about ARI signs/symptoms during the preceding week, and assessed symptomatic children for World Health Organization-Integrated Management of Childhood Illness (WHO-IMCI) danger signs including: tachypnea, wheezing, retractions, grunting, nasal flaring, stridor, or cyanosis [9]. If the previous weekly visit was missed, data was collected for the preceding fourteen days, but no further.

### IHIP-Peru community trial

The Integrated Home-based Intervention Package (IHIP) trial was a community-cluster randomized controlled trial conducted in San Marcos Province from 2008–2010, that randomized 51 rural communities to either an intervention (25 communities, 267 households) or control arm (26 communities, 267 households) [10]. At baseline, all homes used open indoor fires for cooking. The intervention included construction of improved, vented stoves, kitchen sinks with running water, the promotion of hand washing, and installation of solar drinking water disinfection units. Control households received a mother-child psychomotor stimulation intervention. The IHIP trial enrolled one child (aged 6 to 35 months) per household and children were followed from February 2009 to January 2010. Primary outcomes were the frequency of diarrhea and acute lower respiratory tract infections (ALRI) in enrolled children [11], [12]. Children who participated in the IHIP trial were eligible for subsequent enrollment in the RESPIRA-PERU study if they met enrollment criteria [8].

### Nasal swab samples

Workers collected nasal swabs (NS) for each ARI episode at the first household visit following onset of symptoms as previously described [6], [8], [13]. After collection and processing, samples were shipped on dry ice to Vanderbilt University for detection of the following respiratory viruses using real-time monoplex RT-PCR as previously described: [6], [14]–[16] influenza, respiratory syncytial virus (RSV), human metapneumovirus (MPV), parainfluenza viruses 1–3 (PIV1–3), human rhinovirus, and adenovirus. For the purposes of this study only results of testing for influenza viruses (A, B, and C) will be presented.

### Statistical analysis

All statistical analyses were performed using Stata 12.1 (StataCorp, College Station TX).

### Incidence calculation

Crude influenza incidence was calculated as the number of influenza-positive ARI episodes, divided by the cumulative person-time under observation and at risk. Children were not considered at risk during an ARI or within 7 days of ARI resolution. Crude estimates were adjusted to account for recurrence of events within individuals (some children had more than one confirmed influenza episode) using zero-inflated negative binomial regression. Poisson regression was used to calculate incidence for each influenza type.

### Comparison of clinical features

The demographic and clinical features of influenza-associated ARI, other viral ARI, and ARI in which no virus was detected, were compared using medians and proportions. A mixed-effects logistic regression model was used to test for significant differences, accounting for the recurrence of ARI at the level of the individual child.

### Risk factors for influenza infection

Multivariable, mixed-effect Poisson regression modeling, accounting for correlation of influenza infection at the level of the individual child was used to evaluate potential risk factors for influenza illness. Only 279 of the 892 children enrolled in RESPIRA-PERU (31%) had received any influenza vaccination at any time prior to study end; vaccination status was therefore not included in the risk factor analysis. Because risk for influenza infection existed only when influenza was present in the community, the analysis was limited to months during which influenza was circulating, defined as any month during which more than one influenza-positive NS specimen was identified among study children. To minimize misclassification the analysis was further restricted to children already under observation at the start of each influenza season, since children entering observation during the influenza season may have experienced an influenza ARI prior to observation onset.

## Results

### Influenza type, subtype, and incidence

**Figure 1:**
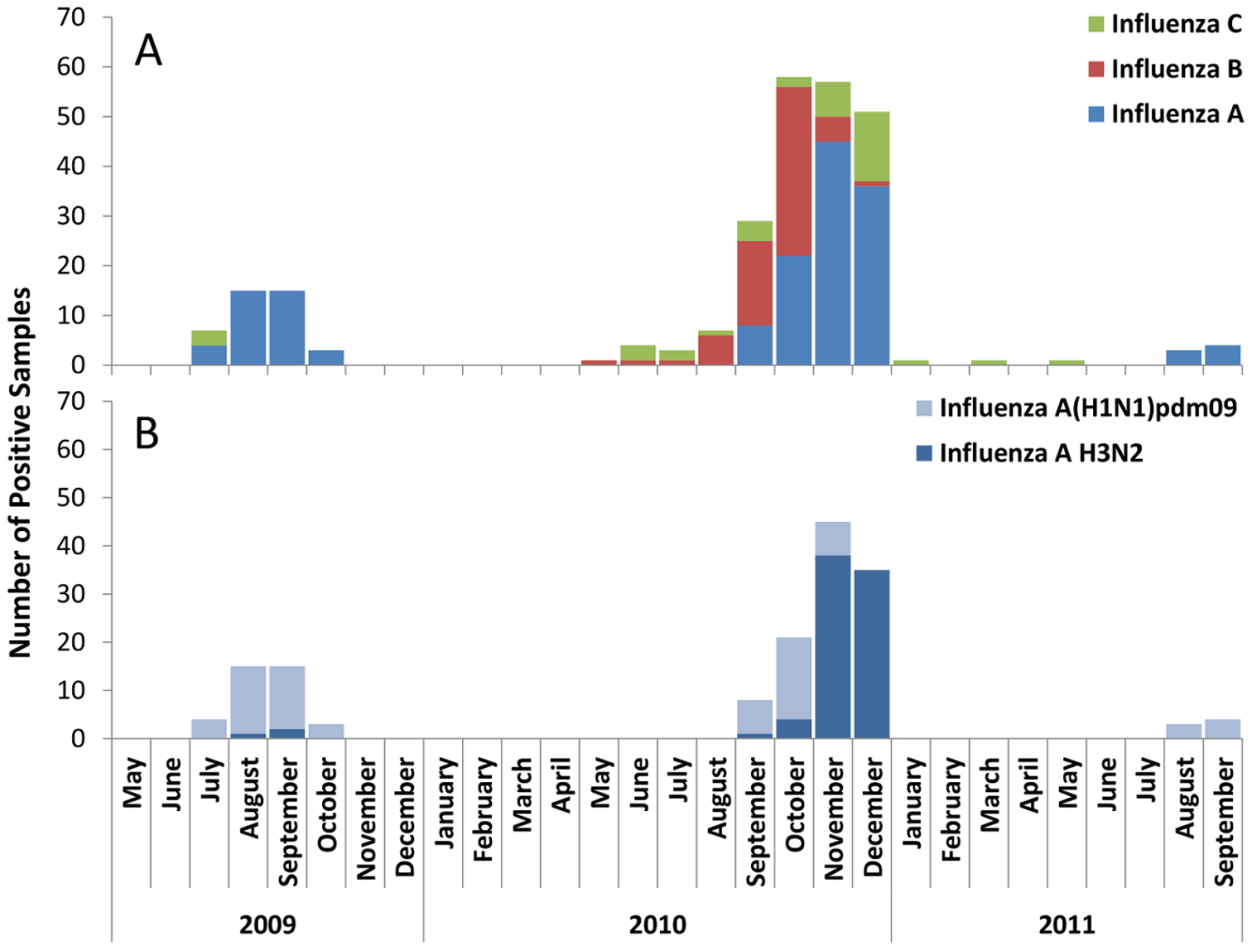
Seasonality of influenza-associated ARI.

The RESPIRA-PERU study enrolled 892 children from 810 households in 58 communities. These children experienced 4,475 symptomatic ARI episodes over the course of the study and NS specimens were collected for 3957 (88%) [7]. Influenza was detected in 258 NS specimens, with the majority of influenza-positive ARI occurring in 2010, when all three types (A, B, and C) co-circulated (Figure 1A). Incidence of all influenza episodes was 36.6 per 100 child-years (Table 1); because the study contained three influenza seasons but not three complete calendar years, this may overestimate the true annual risk. Monthly influenza incidence ranged from 0 to 112 influenza-positive ARI per 100 child years and was highest in October – December (Figure 1, Table S1). Subtype A(H1N1)pdm09 was the predominant isolate in 2009 and 2011, while seasonal H3N2 virus was predominant in 2010 (Figure 1B). Among the 155 influenza A infections, 72 (46%) were A(H1N1)pdm09, 81 (52%) were H3N2, and 3 (2%) were indeterminate. There were 35 children with two influenza-positive ARI during the study period, and one child who had three. All of the children with two or more influenza-positive ARI experienced their second (or third) infection in 2010; the vast majority also had their first infection in 2010 (Table 2). Reinfection with the same type was rare. Five children experienced multiple influenza A episodes; three contracted both A(H1N1)pdm09 and H3N2 infections in the 2010 influenza season, the other two had A(H1N1)pdm09-associated ARI in 2009 and H3N2-associated ARI in 2010. Two children had recurrent influenza B-associated ARI; each had their first influenza-positive ARI in September, 2010 and a second influenza B-associated ARI in October, 2010, potentially indicating prolonged shedding rather than new infection. Co-detection of non-influenza study viruses occurred in 71 (28%) of the 258 influenza-associated episodes; among these were 54 (76%) human rhinovirus, 22 (31%) adenovirus, and 15 (21%) PIV1-3 coinfections, and one (1%) RSV co-infection.

**Table 1:** Influenza incidence per 100 child-years.

Influenza type and subtype	Cases	Incidence (95% CI)
Influenza A (all subtypes)	155*	20.5 (17.5–24.0)
A(H1N1)pdm09	72	9.5 (7.6–12.0)
H3N2	81	10.7 (8.6–13.3)
Influenza B	66	8.7 (6.9–11.1)
Influenza C	39	5.2 (3.8–7.1)
Total	258†	36.6 (31.0–43.3)

*One sample tested positive for both pandemic H1N1 and H3N2 and 3 were untypable.

† Total does not equal sum of A+B+C because 2 samples contained more than one influenza strain.

**Table 2:** Number of Children Experiencing Multiple Influenza Episodes.

	Second influenza virus detected (calendar year 2010)*				
First Influenza Virus	A(H1N1)pdm09	H3N2	Influenza B	Influenza C	Total
**Calendar Year 2009**					
Influenza A(H1N1)pdm09	0	2	1	2	5
Influenza A H3N2	0	0	0	0	0
Influenza B	0	0	0	0	0
Influenza C	0	1	0	0	1
**Calendar Year 2010**					
Influenza A(H1N1)pdm09	0	3	0	2	5
Influenza A H3N2	0	0	0	5	5
Influenza B	2†	13	2†	0	17
Influenza C	0	1	3	0	4

*All second infections occurred in calendar year 2010.

† Two children had influenza B detected twice during 2010; one of these children also subsequently had influenza A(H1N1)pdma09 detected the same season.

### Characteristics of influenza-associated ARI

The characteristics of influenza ARI, non-influenza viral ARI, and ARI in which no study virus was identified are compared in Tables 3 and 4. Influenza illness was more common in 2010. A higher proportion of influenza ARI occurred in the 2^nd^ altitude quartile, and fewer in the 4^th^, compared to other ARI (Table 3). There were no significant differences in symptom duration, presence of WHO-IMCI danger signs, or hospitalization rates between influenza types A and B, but influenza type C-associated ARI had significantly fewer days of fever and malaise (Table 4). ARI positive for non-influenza viruses also had fewer days fever and malaise, as well as proportionally fewer healthcare visits, compared to influenza A. Influenza A or B-associated ARI had more days of symptoms and were more likely to prompt a healthcare visit than other ARI (Table 4).

**Table 3:** Child characteristics associated with each symptomatic ARI episode that was tested for the presence of respiratory viruses.

Characteristic	Influenza ARI (N = 258)	Non-influenza viral ARI (N = 2390)	Other ARI (N = 1309)
Male	49.2 (43.1–55.3)	52.0 (50.0–54.0)	52.7 (50.0–55.4)
Age in months, mean (95% CI)	16.3 (15.1–17.5)	15.8 (15.4–16.2)	**14.1 (13.6–14.7)** *
Calendar year			
2009	15.5 (11.1–19.9)	18.7 (17.2–20.4)	**26.4 (24.0–28.8)** *
2010	80.6 (75.8–85.4)	**44.4 (42.4–46.4)** *	**48.4 (45.7–51.1)** *
2011	3.9 (1.5–6.2)	**3.67 (3.59–3.87)** *	**2.51 (2.27–2.75)** *
Observation days, mean (95% CI)	425 (406–446)	**404 (397–410)** *	408 (399–417)
Shares a bed	97.3 (95.3–99.2)	97.2 (96.6–97.9)	96.1 (95.1–97.1)
Attends daycare	6.6 (3.6–9.7)	7.4 (6.3–8.4)	6.3 (5.0–7.6)
Household conditions			
Persons per household, median (IQR)	4 (5–6)	4 (5–6)	4 (5–6)
Children <5 years old per household, median (IQR)	1 (1–2)	1 (1–2)	1 (1–2)
Number of rooms in dwelling, median (IQR)	2 (2–3)	2 (2–3)	2 (2–3)
Electricity in home	39.9 (33.9–45.9)	41.1 (39.2–43.1)	43.1 (40.5–45.8)
Water from pipeline or well	84.1 (79.6–88.6)	85.0 (83.6–86.5)	86.5 (84.6–88.3)
Municipal sewer or septic tank	17.8 (13.2–22.4)	20.6 (19.0–22.2)	19.1 (17.0–21.2)
Wood fuel used for cooking	95.3 (92.8–97.9)	95.2 (94.3–96.0)	93.4 (92.0–94.7)
Presence of smoker in home	9.6 (6.0–13.3)	11.1 (9.8–12.4)	10.3 (8.6–11.9)
Father a laborer	80.6 (75.8–85.4)	82.0 (80.4–83.5)	80.7 (78.6–82.9)
Mother did not complete secondary education	89.8 (86.1–93.5)	88.5 (87.2–89.8)	87.1 (85.3–89.0)
% Distribution in each altitude quartile			
Q1) 1976–2321 meters (N = 195 children)	23.6 (18.4–28.8)	27.2 (25.5–29.1)	28.0 (25.5–30.4)
Q2) 2322–2641 meters (N = 189 children)	31.4 (25.7–37.1)	**23.2 (21.5–24.9)** *	**24.4 (22.0–26.7)** *
Q3) 2643–2869 meters (N = 199 children)	26.7 (21.3–32.2)	24.4 (22.7–26.1)	26.2 (23.8–28.6)
Q4) 2870–3803 meters (N = 189 children)	18.2 (13.5–22.9)	**25.1 (23.3–26.8)** *	21.5 (19.2–23.7)
Household in IHIP trial†			
No, open stove (N = 376 children)	60.8 (54.9–66.8)	58.0 (55.0–59.2)	57.1 (54.5–59.8)
No, improved stove (N = 133 children)	14.7 (10.4–19.1)	16.3 (14.8–17.8)	17.6 (15.6–19.7)
Yes, control arm (N = 136 children)	15.9 (11.4–20.4)	14.6 (13.2–16.1)	13.1 (11.3–15.0)
Yes, intervention arm (N = 127 children)	8.5 (5.1–11.9)	11.8 (10.5–13.1)	12.1 (10.3 –13.8)

Data represent percentages (95% CI) unless otherwise indicated.

*P<0.05 for strength of association in logistic regression model, adjusting for correlation at the level of the individual child.

† In total, 772 (86.5%) of the 892 children enrolled in the study had at least one ARI for which a NS sample was tested.

**Table 4:** Clinical attributes of influenza-associated acute respiratory illnesses (ARI) episodes vs. non-influenza episodes among ARI episodes for which nasal swab samples were collected.

Attribute	Influenza A (N = 153)	Influenza B (N = 64)	Influenza C (N = 39)	All Influenza †(N = 258)	Non-influenza viral ARI (N = 2,390)	Other ARI (N = 1,390)
Age in months, mean (95% CI)	15.9 (14.3–17.4)	17.6 (14.9–20.4)	16.5 (13.1–19.9)	16.3 (15.1–17.5)	15.8 (15.4–16.2)	14.1 (13.6–14.7)*
Male (%)	49.7 (41.8–57.6)	39.1 (27.1–51.0)	64.1 (49.0–79.2)	49.2 (43.1–55.3)	52.0 (50.0–54.0)	52.7 (50.0–55.4)
Days with symptoms, median (IQR)	6 (4–9)	7 (4.5–9.5)	6 (4–10)	6.5 (4–9)	5 (3–9)	4 (2–8)*
Fever	2 (1–4)	2 (1–4)	1 (0–2)*	2 (1–3)	1 (0–2)*	2 (1–3)*
Cough	5 (3–8)	6 (3–8.5)	5 (3–10)	5 (3–9)	5 (2–8)	3 (0–6)*
Malaise	2 (0–4)	2.5 (0–5)	0 (0–2)*	2 (0–4)	0 (0–2)*	0 (0–2)*
Rhinorrhea	5 (3–8)	6 (3–9)	5 (3–9)	5 (3–8)	4 (2–7)	2 (0–5)*
Exam for severity performed (%)	67.3 (59.9–74.8)	64.1 (52.3–75.8)	74.4 (60.7–88.1)	67.1 (61.3–72.8)	65.6 (63.7–67.5)	44.2 (41.5–46.9)*
WHO-IMCI danger signs present (%)	6.8 (1.9–11.7)	7.3 (0.0–15.3)	6.9 (0.0–16.1)	6.9 (3.2–10.7)	8.0 (6.7–9.4)	6.2 (4.3–8.2)
Healthcare visit (%)	36.6 (29.0–44.2)	46.9 (34.6–59.1)	25.6 (11.9–39.3)	37.6 (31.7–43.5)	23.3 (21.7–25.0)*	24.1 (21.8–26.5)*
Hospitalization (%)	0.7 (0.0–1.9)	3.1 (0.0–7.4)	0	1.2 (−0.1–2.3)	1.0 (0.6–1.5)	0.6 (0.2–1.0)

*P value vs. Influenza A<0.05 using mixed effect logistic regression to account for >1 ARI episode per child.

† Total does not equal sum of A+B+C because 2 samples contained more than one influenza strain.

### Risk factors for influenza illness

Aside from calendar year, the only factor significantly associated with reduced influenza incidence in multivariable modeling was household participation in the IHIP community trial (Table 5). Compared to children from households not using improved stoves and not in the IHIP trial, children in IHIP intervention households had a relative risk (RR) for influenza-associated illness of 0.35 (95% CI 0.19–0.62), while children living in IHIP control households had a RR of 0.61 (95% CI 0.37–0.98).

**Table 5:** Risk factors for influenza incidence among children under observation during months of influenza circulation.

	Univariate analysis		Multivariate analysis	
	Relative risk (95% CI)	P value	Relative risk (95% CI)	P value
Age	1.00 (0.98–1.01)	0.655	1.01 (1.00–1.03)	0.158
Male gender	0.96 (0.73–1.26)	0.759	0.95 (0.72–1.26)	0.732
Calendar year				
2009	reference		reference	
2010	**2.46 (1.55–3.91)**	**<0.001**	**1.80 (1.07–3.01)**	**0.025**
2011	**0.28 (0.12–0.67)**	**0.004**	**0.16 (0.06–0.41)**	**<0.001**
Crowding				
Persons per room	0.95 (0.87–1.05)	0.342	0.99 (0.89–1.09)	0.815
Child shares a bed	1.20 (0.49–2.91)	0.688	1.17 (0.47–2.88)	0.736
Attends day-care	1.12 (0.67–1.86)	0.671	1.07 (0.63–1.81)	0.800
Physical environment				
Water from well or pipeline	0.90 (0.62–1.30)	0.581	0.93 (0.63–1.36)	0.690
Municipal sewer or septic tank	0.79 (0.55–1.14)	0.215	0.88 (0.60–1.29)	0.513
Electricity	1.01 (0.76–1.33)	0.967	0.96 (0.68–1.34)	0.793
Wood primary cook fuel	1.44 (0.74–2.82)	0.281	1.11 (0.52–2.34)	0.79
Altitude quartile				
1) 1976–2321 meters	reference		reference	
2) 2322–2641 meters	1.34 (0.92–1.97)	0.132	1.29 (0.86–1.94)	0.212
3) 2643–2869 meters	1.13 (0.77–1.66)	0.539	1.23 (0.81–1.87)	0.337
4) 2870–3803 meters	0.83 (0.55–1.26)	0.385	0.88 (0.55–1.42)	0.605
Household participation in IHIP trial				
Non-participant, open stove	reference		reference	
Non-participant, improved stove	0.70 (0.47–1.06)	0.09	0.73 (.047–1.14)	0.169
Participant, control arm	0.72 (0.49–1.07)	0.107	0.61 (0.37–0.98)	0.042
Participant, intervention arm	**0.43 (0.26–0.71)**	**0.001**	**0.35 (0.19–0.62)**	**<0.001**

### Subgroup analysis of IHIP trial households

A separate subgroup analysis including only children who had been in the IHIP trial (N = 105 from intervention households and 112 from control households) showed 11 influenza ARI over 32.5 child-years among children from intervention households and 26 influenza ARI over 34.3 child-years among children from control households. In multivariable analysis including calendar year, age, and gender, children in IHIP intervention households had a RR for influenza illness of 0.40 (95% CI 0.20–0.82), compared to children from control households.

## Discussion

Influenza is increasingly recognized as an important cause of severe respiratory infection in children worldwide [3]–[6], [17], [18]. The incidence of influenza illness among RESPIRA-PERU cohort children was high, with rates of 20.5, 8.7 and 5.2/100 child-years for influenza A, B and C, respectively. ARI positive for influenza A or B lasted longer, had more days of fever and malaise, and resulted in more healthcare encounters than non-influenza ARI.

The incidence of influenza ARI observed in RESPIRA-PERU (36.6/100 child-years) is higher than seen in recent household-based studies in other developing nations. A study in Dhaka, Bangladesh from 2004 to 2007 estimated an annual influenza risk of 10% among children <5 years of age, using viral culture to document influenza infection among children with fever or respiratory symptoms [4]. The use of RT-PCR rather than culture in our cohort, and seasonal variation in influenza circulation are factors that may contribute to the higher rates observed in our study. Like our study, the Dhaka study found more fever and malaise among influenza than non-influenza ARI.

A study in Vietnam from 2007–2010 reported a seasonal risk of influenza infection of 41.8% among children aged 5–9 years (no similar data for younger children were reported), using a combination of serology and RT-PCR of NS samples [19]. Despite active weekly surveillance, the Vietnam study found that only 11–16% of laboratory-confirmed influenza infections correlated with an episode of influenza-like-illnesses (ILI, defined as fever plus either cough or sore throat), suggesting that many influenza infections did not result in typical ILI symptoms. Because our study did not employ serologic testing it is unclear how many influenza infections escaped detection by symptomatic surveillance; however, the proportion of undetected infections is likely to be lower for several reasons. First, a more sensitive ARI definition (fever or cough) was used. Of the 258 influenza ARI in our study, 70 (28%) had either fever or cough, but not both, and thus may not have met the Vietnam study definition for ILI (our study did not collect data on sore throat). Second, a higher proportion of influenza episodes in our study were due to pandemic A(H1N1)pdm09, which was more likely to cause symptoms among young children [18]. Finally, both studies relied on parental report of fever and the Vietnam study authors speculate there may have been under-reporting of fever by parents in their study [19].

Some important aspects of our study should be noted when comparing the results to other studies. RESPIRA-PERU was designed to capture all ARI episodes among cohort children and NS samples were collected only once per episode. Recurrence of fever or cough within 7 days of an antecedent ARI episode was considered an extension of the prior ARI, and not counted as a new episode. Thus, children were not considered at-risk for a new ARI during a symptomatic episode and for seven days thereafter, and this time not-at-risk was excluded from the person-time denominator. On average, children in RESPIRA-PERU experienced 6 ARI/year, lasting approximately five days each [7]. Therefore, our exclusion of person-time not at-risk yielded an influenza incidence estimate approximately 26% higher than if time not at-risk had been included. In addition, our surveillance period included three influenza seasons (2009–2011), but only 29 total months, potentially enriching our observation period with time when influenza was circulating. Thus, our influenza ARI incidence estimates, while accurate for the months observed, may overestimate the true annual risk.

Multiple aspects of the physical environment in which children in the RESPIRA-PERU cohort live may affect their susceptibility to respiratory infection. While living in a remote location might insulate households from circulating infections [20], harsh winters may cause indoor crowding and increased exposure to household air pollution. A potential protective effect of remote location during the 1918 influenza epidemic has been suggested [21], yet even the most remote locations are unlikely to be insulated from influenza transmission in the modern era of global mobility. Indeed, influenza caused substantial morbidity in the communities in the RESPIRA-PERU study despite their remote location. Although we did not assess the social interactions or contact patterns during influenza epidemics, additional information on this subject would be useful. Study communities had low population density (about 40 persons per km^2^), yet the high incidence of influenza in our study compared to that observed in highly crowded Bangladesh [3], [5] does not suggest any protective effect of low population density.

Previous studies have shown that Andean Peruvian children are at higher risk for hypoxia with ARI [22], and have higher ARI-related mortality than children in lower-altitude provinces [23]. We observed no association between altitude quartile and risk of influenza-associated ARI in RESPIRA-PERU. Interestingly, the odds of both hospitalization and death among patients with laboratory confirmed influenza A(H1N1)pdm09 in Mexico increased with increasing altitude quintile up to 1765 meters, but did not increase between the fourth (1765–2229 meters) and fifth (2230–2850 meters) quintiles, suggesting the effect of altitude may plateau at very high elevations [24]. Perhaps we observed no effect of altitude in the current study because all communities in San Marcos are located above 1900 meters.

Only 4% of influenza-associated ARI in the RESPIRA-PERU study met WHO-IMCI criteria for severity, a proportion lower than for RSV, MPV, PIV 1–3, and even adenovirus [7]. This may be because fever and malaise, rather than respiratory distress or wheezing, were the distinguishing features for influenza-associated illness in our study. The proportion of influenza episodes prompting healthcare visits, a surrogate for the perceived severity of infection, was 37% and 47% for influenza A and B, respectively, percentages equaled only by RSV among viral causes of ARI [7]. ARI associated with influenza A or B detection were also longer and had more days fever and malaise than ARI associated with other viruses and those from which no viruses were detected. Therefore, the low percentage of influenza-ARI classified as severe in this study should not be misinterpreted as suggesting low morbidity.

The association between enrollment in the IHIP trial [10] and decreased risk for influenza is intriguing, but difficulty to interpret. No significant difference in overall ALRI between communities randomized to intervention or control households was observed in the IHIP trial (Hartinger et al., unpublished data), yet the IHIP trial did not ascertain ARI etiology. Our observation that children in both arms of the IHIP trial had a lower risk of influenza-ARI than children from households not participating in the trial suggests that some factor common to both groups (perhaps enhanced general hygiene awareness secondary to IHIP trial participation) contributed to this finding. Although age and calendar year were obvious potential confounders, the association remained significant after adjustment for these variables. Importantly, when our analyses were restricted to the subset of children that participated in the IHIP trial, those in the intervention arm had significantly lower influenza rates than those in the control arm. Whether this is due to a specific component of the intervention, the combined effect of multiple components or to other factors is unknown. Hand washing, one component of the IHIP intervention, has previously been shown to reduce ARI and pneumonia incidence in low-resource settings [25].

Our study has some important limitations. First, we did not employ serologic testing and NS samples were taken only during symptomatic ARI episodes. As discussed above, several serologic studies suggest that not all influenza infections are symptomatic enough to be detected by active ARI surveillance [19], [26]–[28]. This may have led to misclassification of some children in our risk factor analysis. However, there is no reason to suspect differential misclassification according to household participation in the IHIP trial. To reduce the potential for misclassification we limited our risk factor analyses to months during which influenza was circulating and to children under observation at the onset of each influenza season. Of note, removing these restrictions (i.e. examining influenza cases detected at any time and in all enrolled children) yielded equivalent results (relative risk of influenza 0.26 [95% CI 0.16–0.44] among IHIP intervention households and 0.49 [95% CI 0.32–0.75] among IHIP trial control households). Second, the inherent seasonal variation in influenza limits our ability to draw general conclusions about influenza burden in Andean children, particularly since a large proportion of cases were influenza A(H1N1)pdm09, which disproportionately affected young children [18]. However, comparison to studies in other locations suggests that influenza burden among Andean children is among the highest reported for concurrent influenza seasons [3], [5], [19]. Third, as discussed above, the low percentage of influenza-associated ARI categorized as severe in our study likely underrepresents the perceived morbidity caused by influenza. Finally, we were unable to conduct any analysis of influenza vaccine effectiveness in this population, as less than a third of study children received an influenza vaccine at any time, and much of that use was seasonal vaccine rather than pandemic influenza vaccine.

In conclusion, the incidence of influenza-associated illness among Peruvian Andean children during the 2009–2011 influenza seasons was high. Influenza type A and B infections resulted in more days with fever and malaise and a higher rate of care-seeking than ARI from which other viruses or no virus was detected. Given the low vaccination coverage among cohort children, a substantial amount of respiratory morbidity in Andean children might be averted by universal influenza vaccination. In addition, we found that a home-based intervention targeting home hygiene and indoor air pollution was associated with a substantial reduction in influenza ARI risk among children who participated in the IHIP trial. This suggests an additional role for environmental modifications in prevention of influenza morbidity in this setting.

## Supporting Information

Table S1 Influenza Incidence among Andean Peruvian Children 2009–2011 by Calendar Month.
(DOCX)
