# Correction: Impact of Home Environment Interventions on the Risk of Influenza-Associated ARI in Andean Children: Observations from a Prospective Household-Based Cohort Study

**DOI:** 10.1371/journal.pone.0099689

**Published:** 2014-06-02

**Authors:** The PLOS ONE Staff

The thirteenth author’s name is spelled incorrectly. The correct name is Carlos G. Grijalva. The publisher apologizes for this error.

## References

[1] Budge PJ, Griffin MR, Edwards KM, Williams JV, Verastegui H, et al (2014) Impact of Home Environment Interventions on the Risk of Influenza-Associated ARI in Andean Children: Observations from a Prospective Household-Based Cohort Study. PLoS ONE 9(3): e91247 doi:10.1371/journal.pone.0091247 24622044 10.1371/journal.pone.0091247 PMC3951509

