# Supplementary material for: Impact of Home Environment Interventions on the Risk of Influenza-Associated ARI in Andean Children: Observations from a Prospective Household-Based Cohort Study
Source: PLoS One. 2014 Mar 12;9(3):e91247. doi: 10.1371/journal.pone.0091247 (PMC3951509; doi:10.1371/journal.pone.0091247)
Supplement: Table S1 — Influenza Incidence among Andean Peruvian Children 2009–2011 by Calendar Month. (DOCX) [file pone.0091247.s001.docx]

| **Table S1: Influenza Incidence among Andean Peruvian Children 2009 - 2011 by Calendar Month** | | | |
| --- | --- | --- | --- |
| Month | Influenza-Associated ARI | Child-Years Observation | Incidence / 100 Child-Years |
| January | 1 | 59.93 | 1.7 (0.2 - 11.8) |
| February | 0 | 54.04 | 0 |
| March | 1 | 58.89 | 1.7 (0.2 - 12.1) |
| April | 0 | 56.85 | 0 |
| May | 2 | 66.63 | 3.0 (0.8 - 12.0) |
| June | 4 | 65.37 | 6.1 (2.3 - 16.3) |
| July | 10 | 68.71 | 14.6 (7.8 - 27.0) |
| August | 25 | 83.36 | 30.0 (20.3 - 44.4) |
| September | 48 | 81.78 | 58.7 (44.2 - 77.9) |
| October | 59 | 52.44 | 112.5 (87.2 - 145.2) |
| November | 57 | 57.20 | 99.7 (76.9 - 129.2) |
| December | 51 | 49.85 | 102.3 (77.8 - 134.6) |
